# Deep Learning-Based Method for Detecting Traffic Flow Parameters Under Snowfall

**DOI:** 10.3390/jimaging10120301

**Published:** 2024-11-22

**Authors:** Cheng Jian, Tiancheng Xie, Xiaojian Hu, Jian Lu

**Affiliations:** 1 Nanjing LES Information Technology Co., Ltd., Nanjing 211189, China; chengjianpaper@163.com; 2 Jiangsu Province Collaborative Innovation Center of Modern Urban Traffic Technologies, Southeast University, Nanjing 211189, China; huxiaojian@seu.edu.cn (X.H.); lujian_1972@seu.edu.cn (J.L.); 3 Jiangsu Key Laboratory of Urban ITS, Southeast University, Nanjing 211189, China; 4 School of Transportation, Southeast University, Nanjing 211189, China

**Keywords:** snow removal, deep learning network, virtual coil, traffic flow parameter estimation, vehicle detection

## Abstract

In recent years, advancements in computer vision have yielded new prospects for intelligent transportation applications, specifically in the realm of automated traffic flow data collection. Within this emerging trend, the ability to swiftly and accurately detect vehicles and extract traffic flow parameters from videos captured during snowfall conditions has become imperative for numerous future applications. This paper proposes a new analytical framework designed to extract traffic flow parameters from traffic flow videos recorded under snowfall conditions. The framework encompasses four distinct stages aimed at addressing the challenges posed by image degradation and the diminished accuracy of traffic flow parameter recognition caused by snowfall. The initial two stages propose a deep learning network for removing snow particles and snow streaks, resulting in an 8.6% enhancement in vehicle recognition accuracy after snow removal, specifically under moderate snow conditions. Additionally, the operation speed is significantly enhanced. Subsequently, the latter two stages encompass yolov5-based vehicle recognition and the employment of the virtual coil method for traffic flow parameter estimation. Following rigorous testing, the accuracy of traffic flow parameter estimation reaches 97.2% under moderate snow conditions.

## 1. Introduction

The application of intelligent traffic surveillance systems (ITSSs) is more advantageous than traditional traffic monitoring systems. Compared with traditional monitoring systems, ITSSs have been considered more effective and helpful in detecting the entire traffic flow. Most traditional traffic monitoring devices, such as induction coil and radar, can only obtain fixed and discrete traffic location information. In contrast, ITSSs can capture continuous traffic flow using roadside and car cameras. Furthermore, the camera images will cover all the information about traffic flow, including vehicle class, vehicle license, and even the situations that cause vehicles to change lanes, which is 3-D information. Also, the advancements in image processing have yielded significant benefits in enhancing the accuracy of surveillance video images beyond the capabilities of conventional devices.

Consequently, these advancements have found widespread application in areas such as illicit vehicle capture and vehicle safety distance detection. As an intelligent system, ITSSs can also provide data about the real-time road congestion situation and incidents where the camera has been placed for surveillance of vehicle information. Above all, ITSSs can provide more accurate and real-time vehicle 3-D information with lower cost, which has better robustness than traditional monitoring devices.

There are many problems with current traffic flow video detection. One of the most significant challenges in real-world traffic monitoring is obtaining clean images under snowfall conditions, which has been a widespread issue all across the world. Snowfall seriously interferes with the background information in the image, so when most target-detection methods are applied to the detection of snowy images, the performance will be greatly reduced. To solve this problem, a number of methods have been proposed. For example, Hase and Starik et al. [1,2] removed snow by adopting the temporal median filtering method on the image based on the time-varying characteristics of snow, but this method is only suitable for relatively simple scenes, and it will blur the important edges in complex scenes.

Based on these studies, more and more efforts have been focused on detecting traffic flow parameters by removing snow particles and streaks from images. At present, with the rapid development of computer vision and image processing, methods for removing snowfall from images can be divided into two categories: image prior knowledge and deep learning methods.

The image prior knowledge method is a way to remove snowfall by using the snow’s features. The simple method of snowfall image prior knowledge uses a filter. This kind of method uses a combination of the visual characteristics of snow and filters; it runs faster but does not yield good performance and is difficult to improve further. Another complex image prior knowledge method trains a complete dictionary for the specific content of the image through sparse coding. The specific operation process of this method usually decomposes the image into low-frequency components without snow particles and high-frequency components containing all snow particle information.

The dual-tree complex wavelet transform (DTCWT) [3] is a powerful method for performing image processing and image restoration. This method is now applied to the processing of video images [4,5,6]. Liu [7] and Lu [8] et al. applied DTCWT to image segmentation, and Sun [9] and Jung [10] et al. combined DTCWT with histogram equalization for luminance enhancement of low-luminance images. Inspired by the fact that DTCWT can obtain multi-angle information from images, the DTCWT has been combined with a deep learning network to remove snow particles of different sizes and angles.

Deep learning networks are the most effective methods for removing snowfall from images and have demonstrated better performance in extracting snowfall from images compared to other methods. This method takes traffic flow images in snowy conditions as inputs and trains the model to achieve the purpose of snow removal and traffic flow detection.

Compared with the traditional methods for snow removal and traffic flow detection, deep learning methods have been rapidly developed in recent years [11,12,13,14,15]. Fu et al. [16] improved the denoising performance of the model by extending the Residual Network [17] (ResNet) and leveraging the prior knowledge of the image domain. Compared with the local feature analysis method of snow particles, the deep learning network built with the multi-layer network structure can extract potential features from the image and formulate more robust strategies, such as target detection and tracking, achieving higher detection rates than traditional machine learning models such as convolutional neural networks (R-CNNs) [18], AlexNet, etc.

In this paper, a framework is proposed for obtaining traffic flow parameters from video images under snowfall conditions, utilizing deep learning networks. The goal of this framework is to obtain the parameters of traffic flow operation under snowy weather from snowfall images and provide a data basis for traffic flow control, vehicle distance detection, and so on. It is designed to work for both light snow and moderate snow. This framework can be composed of four stages: The first and second stages are to remove snow particles and snow streaks from the image containing snow, thus achieving the purpose of removing snow from the image and achieving a clear image. The third step is to achieve the purpose of detecting the traffic flow from a clear image using You Only Look Once version 5 (yolov5). The last step is to obtain the traffic flow parameters from the clear video images using the virtual coil method. The results show that the framework used in this paper can effectively remove snow particles and snow streaks from snow-containing images for the purpose of vehicle detection and traffic flow parameter detection. In other words, using this framework, it is also possible to detect other objects in snowfall weather effectively.

## 2. Literature Review

In order to accurately detect traffic flow in snowy weather, many methods have been proposed in existing research, namely local feature analysis methods of snow referred to as image prior knowledge methods and deep learning methods. The snow feature analysis method refers to the analysis of snow features in the image, thus effectively separating snow and achieving the purpose of traffic flow detection. At present, many methods have been proposed to remove snow from video images based on the image features of snow.

Garg et al. [19] measured the direction of the raindrops and snowfall to improve the accuracy of the method. The method assumes that the raindrops and snowfall are all ellipses and judges the raindrops and snowfall by detecting the direction of the ellipse in the image. However, snow does not have a regular shape, so this method cannot be used for snow removal. Barnum et al. [20] proposed a method to detect snow and rain based on the frequency domain of images by extracting frequencies similar to snow and representing them in the frequency space, achieving the purpose of extracting snow. However, the method is only efficient on rainy days. This is because snow particles, unlike raindrops and rain streaks, can have different sizes and orientations.

Pei et al. [21] constructed an algorithm to remove snow streaks from images by using the image features of saturation and visibility of snow streaks. This method can effectively remove snow streaks from images, but it is not effective for the other two cases due to the lack of snow particles and snow fog in the dataset. Rajderkar et al. [22] used a combination of bilateral filtering and sparse coding to remove snow particles from the snowy part. Xu et al. [23] combined a kinetic model with a photometric model of physics to remove snow particles by determining their motion characteristics, which can remove snow particles to some extent but also blurs the details in the image. Sun et al. [24] used a fuzzy similarity function to construct an algorithm to accurately find the location of rain and snow in the image, which can effectively detect raindrops and snow particles with different velocities but cannot remove the snow fog in the image. Liu [25] used the difference in brightness of pixel points in multi-frame images and applied the improved K-means algorithm to remove snow particles, but the processed result produced a line of snow particles with high brightness, resulting in the loss of a large amount of image information. Hu [26] downscaled the data projection in the RGB three-dimensional space of video images to show the clustering characteristics of high-dimensional data by studying the distribution of low-dimensional data to remove snow.

Compared with the traditional snow removal and traffic flow detection methods, deep learning methods have been rapidly developed in recent years. Bossu [27] et al. used a mixed Gaussian model to separate the foreground and background of the image, and further used the stripe direction histogram to remove snow particles in the foreground, but this method was prone to wrongly identifying traffic flow as the background, and the detection effect of moving targets was not good. Wang et al. [28] constructed a hierarchical approach to decompose snow in pictures into different frequencies and remove them. Liu et al. [29] proposed a snow-removal structure, Desnownet, based on conditional generative adversarial networks and added quantitative criteria to the objective function to improve the performance of snow removal. On this basis, Li et al. [30] further optimized the generative adversarial network to improve the snow-removal performance. Chen [31] et al. added new detection parameters to the snow-removal structure to improve the performance of snow removal and snow fog removal. After that, Chen [32] et al. further used dual-tree complex wavelet transform to remove snow noise in images, which can effectively restore image sharpness. At the same time, Chen et al. also used the method for testing target detection, and the test results show that the method can be effectively applied to target detection in snow.

None of the current snow-removal algorithms [29,30,31,32] with good snow-removal performance using deep learning networks can remove snow streaks from images well, which is caused by the different image features of snow streaks and snow particles. Snow streaks usually have large image pixels, and the training set of the model usually does not contain large pixel snow pattern images because they do not have fixed shape features. The framework used in this paper adopts a distributed strategy to remove snow streaks and snow particles from the images separately. The feature of snow streak sparsity is used for the removal of snow streaks.

From the above literature, it can be seen that the problems of the existing studies are as follows:

- There is a lack of snow processing with traffic flow detection as the goal. Existing research on snowfall weather is mostly oriented to the extraction of snow motion but not for traffic flow detection.
- Existing studies have a relatively weak ability to handle snow streaks, snow particles, and snow fog well and simultaneously. Existing methods mostly target the removal of one aspect of snow characteristics, which limits the robustness of the methods.

This study aimed to improve the accuracy of vehicle detection and obtain traffic parameters under snowy conditions. A framework based on the deep learning network has been proposed, which can remove snow particles and snow streaks from snow-containing images efficiently. The contributions of this paper can be summarized as follows:

- A new framework is proposed that can detect vehicles and traffic flow parameters under snowy weather conditions;
- A deep learning network is incorporated for vehicle detection in traffic surveillance videos;
- A complete framework for traffic flow detection and parameter estimation in real snowfall environments is proposed;
- A new general process is designed for traffic flow detection and parameter estimation in stages one and two.

## 3. Methodology

**A.**  **Overview**

**Figure 1:**
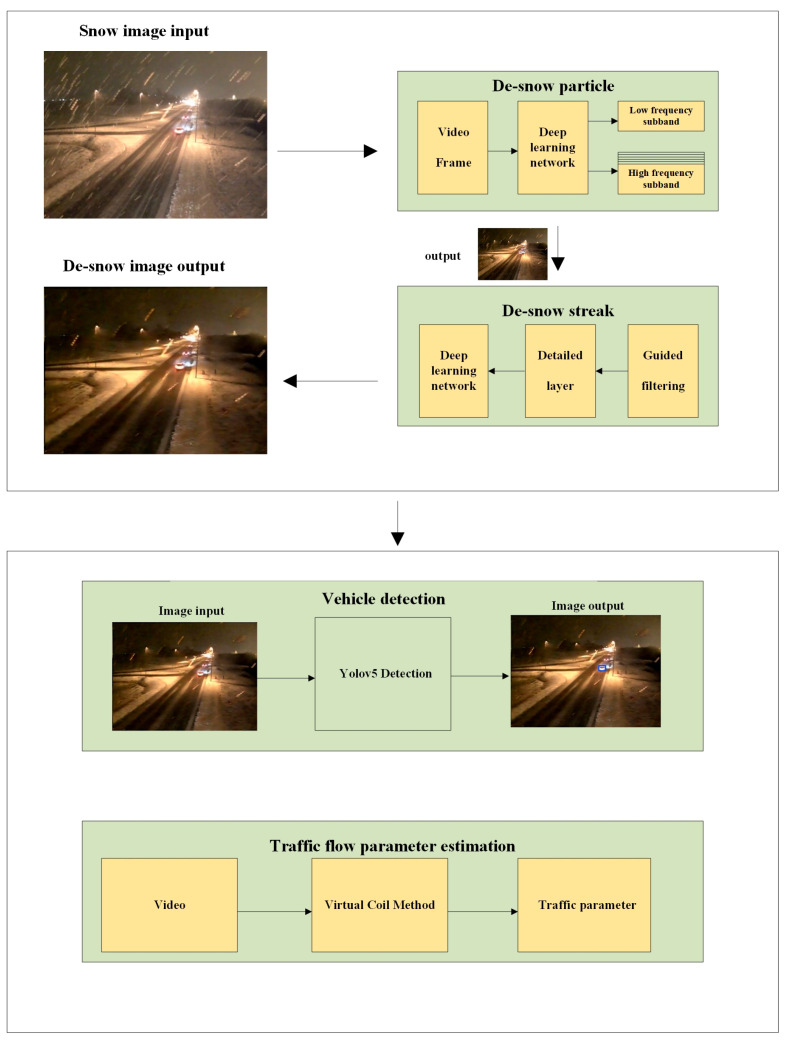
The overview of the framework.

The framework proposed in this paper consists of four stages (see Figure 1). The selection of these four stages is based on a strategic, hierarchical approach that progressively refines the image from basic snow granules to complex traffic flow analysis. Each stage builds upon the previous one, ensuring that the snow interference is effectively managed, and the scene’s true characteristics are revealed. This structured approach allows for a focused and efficient processing pipeline that is tailored to the specific challenges of snow removal and traffic analysis. The function of the first two steps is to remove the snow in the snowy image and the function of the last two stages is to achieve traffic flow detection and parameter estimation. In the first stage, a snow particle removal network has been created based on the DTCWT [3], which can identify snow particles of different sizes and angles efficiently. In the second stage, a deep detail network has been used to remove snow streaks, using the guided filtering method to divide the input image into a detail layer and a base layer. The detail layer is used as the input to the deep detail network, and most of the streak information is included in the detail layer, which can decrease the training time and increase model accuracy [16]. In the third stage, the yolov5 [33] model has been used to detect vehicles in the image. After the first two steps of image processing, using yolov5 in this step can make the detection accuracy almost reach the same standard as on sunny days. In the fourth stage, the speed and volume of the traffic flow is estimated using the virtual coil method, which can estimate the speed and volume of the traffic flow by calculating the time for the vehicle to pass through two adjacent coils. Compared with other parameter estimation methods, the virtual coil method has faster detection speed and smaller memory usage and is widely used for vehicle parameter estimation [34,35]. After the above four steps, the parameters of traffic flow in snowy conditions can be accurately obtained.

**B.**  **Data and Environment Description**

To maximize the snow-removal performance of the model, the dataset used should contain a variety of different snowfall scenarios. The interference of snowfall on the image can be divided into snow particles, snow streaks, and snow fog. In order to train the model and test the accuracy of the model, the dataset should contain these three types of video data. In this paper, the CADC [36], Snow100K [29], AAU [37], and CSD [32] datasets have been used for network training and testing. The CADC dataset is a dataset of real snowfall environment images under autopilot conditions. The Snow100K and CSD datasets are composed of both synthetic and real snowfall images, containing a large number of snow particles, snow streaks, and snow fog images. The AAU dataset contains traffic surveillance camera data from seven different streets and different weather conditions, and has a total of 22 video sequences, including light snow, moderate snow, and heavy snow, which provide a data basis for determining the scope of application of the snow-removal model and testing the accuracy of vehicle recognition for different snowfalls [38]. Regarding the video data, the snow-containing video lasts for a total of 25 min, the video frame rate is 20 frames per second, and the image resolution is 640 × 480.

During the model training, the learning rate of the model is set to 10^−4^ and the optimizer proposed by Adam [39] is applied. This optimizer combines the advantages of both AdaGrad and RMSprop methods; it has the ability to handle sparse gradients and to handle non-smooth targets, and consumes less memory resources, making it well suited for application to model training in deep learning networks. The proposed network model was finally trained on an NVIDIA RTX Titan GPU with epochs set to 1500 and a batch size of 32. For the training data, 10,000 images from the Snow100K and CSD datasets have been used. In each epoch, 30% of the training data were used as the validation set.

The system environment for the tests conducted in this paper is 64-bit Windows, the processor model is an Intel(R) Core (TM) i5-8300H CPU @ 2.30 GHz 2.30 GHz, and the deep learning network used is built based on tensorflow in python, version 2.0, and python, version 3.8. The accuracy of the model is examined using yolov5 as the target-detection model. The yolov5 model used in this paper has been trained using the COCO-128 [40] dataset with a confidence lower limit set to 0.25 and a cross-merge ratio of 0.65.

**C.**  **Stage 1: Snow particle removal network**

In this paper, a model combining DTCWT and Res2net network has been used to construct the snow particle removal network model.

**Figure 2:**
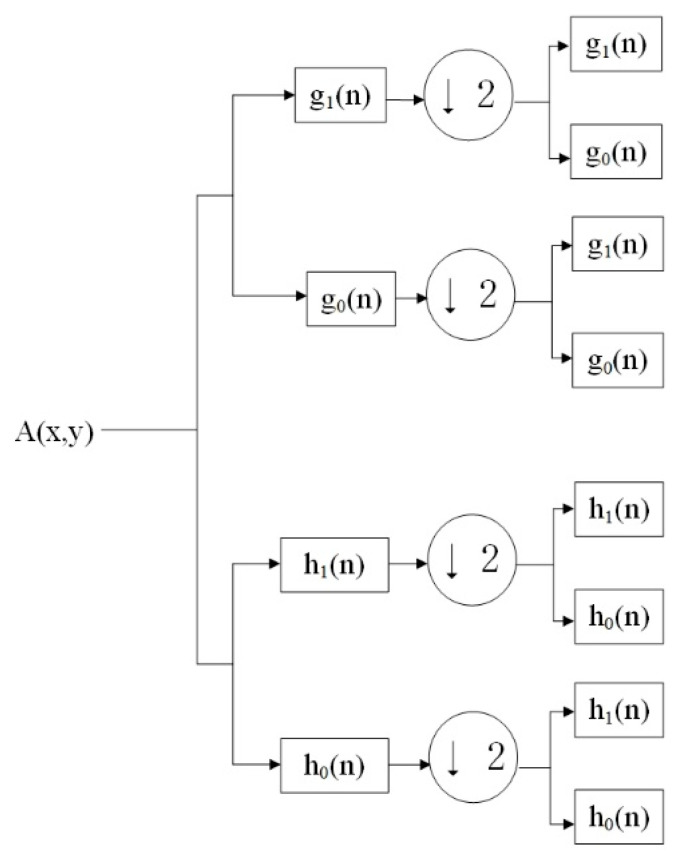
The structure of DTCWT network.

Compared to discrete wavelet transform (DWT) [40], DTCWT has a better orientation selectivity, which makes it more sensitive to geometric edge features. Its network is shown in Figure 2. The snow-containing images input into the DTCWT will be divided into 2 low-frequency sub-bands and 6 high-frequency sub-bands, and the high-frequency sub-bands contain more detailed information in ±15, ±45, ±75, and diagonal directions, respectively. This property can be well applied to remove snow particles of different orientations and sizes.

**Figure 3:**
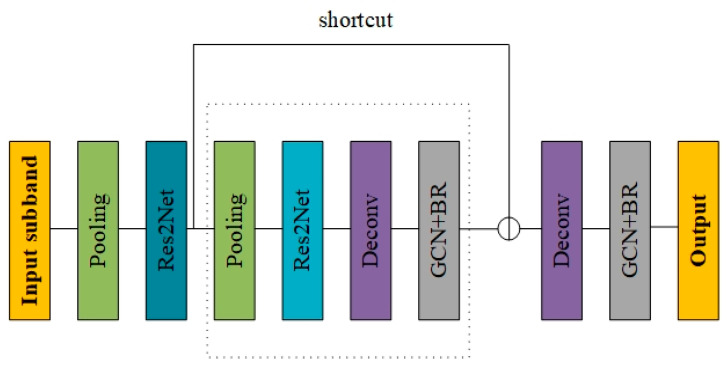
The de-snow particle network.

Res2Net [38] is proposed on the basis of Resnet [41] and shows better performance. Compared with Resnet, Res2net adds smaller residual blocks to the original structure of residual units to increase the size of each layer of the perceptual field of view, and at the same time, Res2Net can be well integrated into other network structures to improve the performance of other networks. The network structure is shown in Figure 3. The snow-containing images are decomposed into six high-frequency images and two low-frequency images after being changed by DTCWT, where each image corresponds to a sub-band. After inputting the sub-band into the model, they first pass through the pooling layer before entering the Res2Net network, which is performed to reduce the number of parameters and computation to prevent the model from overfitting. Using Deconv can effectively reduce the noise in the image and make the feature map smooth. Also, using the image segmentation network GCN+BR after the Deconv layer can effectively improve the performance of the network model [42].

It should be noted that after each sub-band has passed through the network, an inverse wavelet transform is also needed to restore the image. Also, since the lower-frequency sub-bands contain more complex information, processing is performed. The network depth of the low-frequency sub-bands is greater than that of the high-frequency sub-bands, and the convolution kernel size for convolution of the low-frequency sub-bands consists of five types, 2 × 2, 3 × 3, 5 × 5, 7 × 7, and 9 × 9, while the high-frequency sub-bands consist of only 2 × 2, 3 × 3, and 5 × 5.

**Figure 4:**
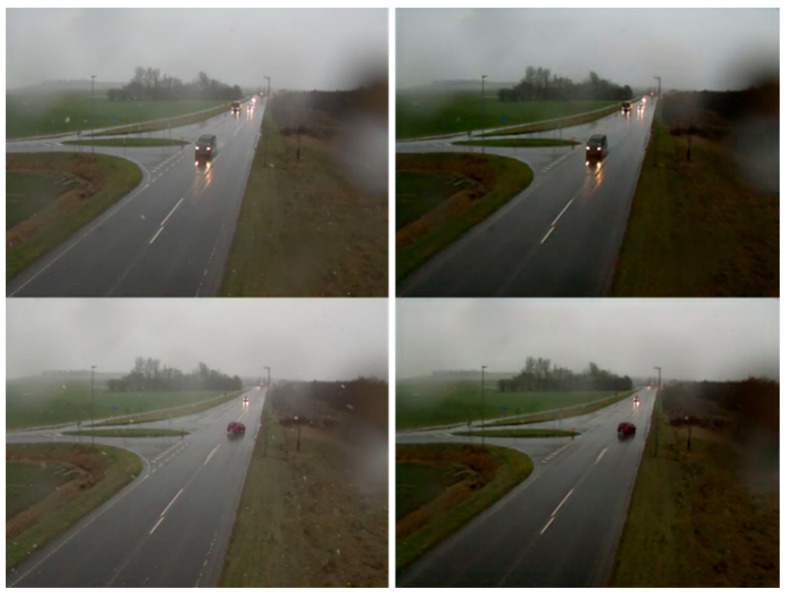
Snow particle removal effect comparison (the left side is the input image; the right side is the output).

The accuracy of the model is further tested using the AAU dataset. The test sample is 30,000 images from the vehicle surveillance video, containing a total of 4300 vehicle samples, and after being processed by the snow-removal model, the images are shown in Figure 4.

**Figure 5:**
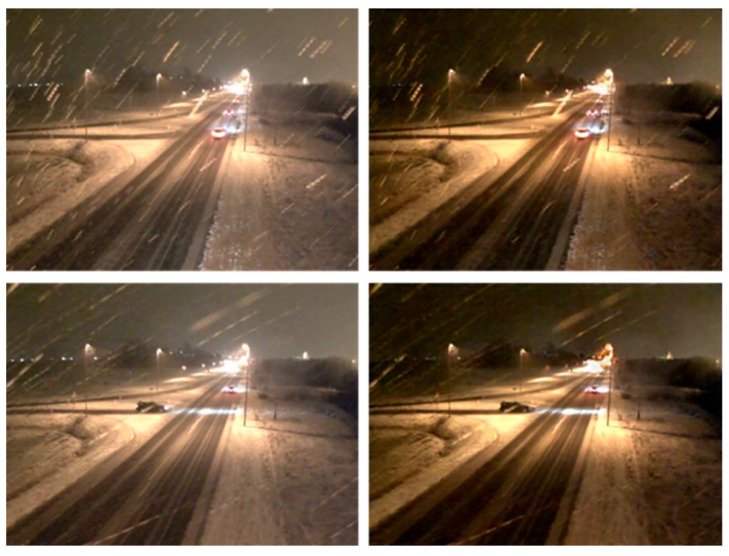
The result of snow removal in the case of snow patterns (the left is the original image; the right is the result).

It can be seen that the correct rate of traffic flow detection in snowfall weather can be effectively improved by using the snow-removal method proposed in this paper. However, the performance when using this method in the presence of snow streaks is poor, which is because the double-tree complex wavelet transform can effectively detect snow particles in different directions, but the lack of snow streaks cannot be detected accurately, as shown in Figure 5.

Therefore, to enhance the accuracy of traffic flow detection in snowfall weather and improve the robustness of the model, further removal of snow streaks from snow-containing images is needed.

**D.**  **Stage 2: Snow streak removal**

The removal of snow streaks in video images is similar to the removal of rain streaks. There are three main methods commonly used to remove rain streaks. The first one is to complete the removal of rain streaks by using the sparsity feature of rain streaks with a priori knowledge of the image. The second method is to use a sparsity dictionary of rain streaks. The sparsity dictionary usually removes other important information such as snow streaks and has a long running time. The third method is the deep learning method, which can express excellent performance with sufficient samples but depends on the features of the training dataset.

**Figure 6:**
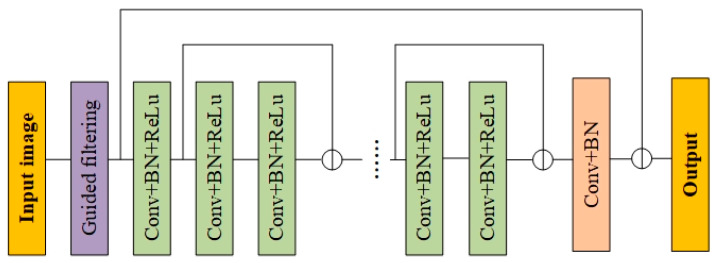
The de-snow streak network.

To address the above problems, the sparsity features of snow streaks have been used as the prior knowledge of the images, the original images have been divided into detail and low-frequency layers by guiding filters, and Resnet and negative mapping have been adopted for the base structure of the model. The network structure is shown in Figure 6.

Due to the combination of the improved Resnet network and the detail layer, the network can learn the features of the snow streaks better than other networks and thus obtain a clean background image. The deep learning network can be roughly divided into two layers. In the first layer, the input image is decomposed into a high-frequency detail layer and a low-frequency layer using the sparsity feature of snow streaks, and then the detail layer image is used as the input to the second layer of the network. In the second layer, the input detail layer image is made to learn the features of snow streaks in the image through the negative mapping network to achieve the purpose of snow streak removal. To prevent the gradient disappearance, BN and RELU have been used after the convolution layer: the BN layer helps the random gradient descent by smoothing the distribution of the input in the hidden layer and mitigates the negative impact of the random gradient descent weight update on the subsequent layers, and the RELU activation function suppresses the negative half of the convolution value and retains the positive half of the convolution value. The use of BN+RELU after the convolution layer can prevent all activation values of a layer from being suppressed, thus preventing all gradients from this layer from becoming 0, i.e., preventing gradients from disappearing. The sparsity of the image means that some coefficients with large values in the image condense most of the details and information in the image; the information of the complete image can be represented using only a small portion of the image. Using this property, complete snow streaks are decomposed into snow layers, and at the same time, the important details in the image are kept in the background layer.

**Figure 7:**
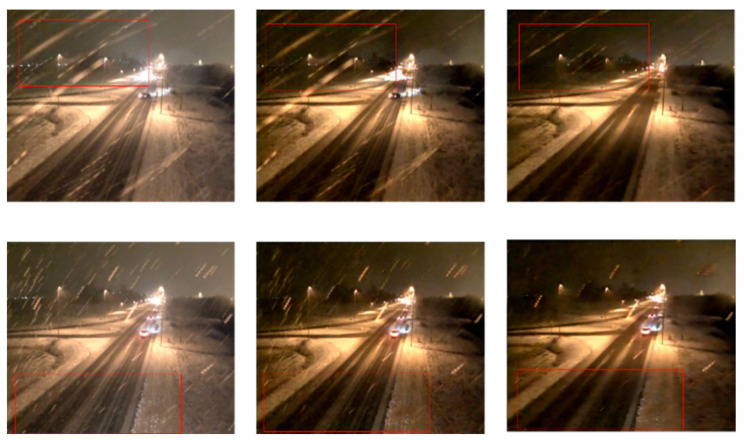
Comparison of results (the left side is the original image, the middle is the de-snow particle model, and the right side is both the de-snow particle model and de-snow streak model).

The snow-containing images were input into the snow particle model, and the model results were used as inputs in the snow streak removal model; the final snow-removal results obtained are shown in Figure 7. The left side is the original image, the middle shows the de-snow particle model output, and the right side is both the de-snow particle model and de-snow streak model. From Figure 7, it can be seen that the final model combining the de-snow particle model and de-snow streak model achieves the best removal results.

**E.**  **Stage 3: Traffic detection**

**Figure 8:**
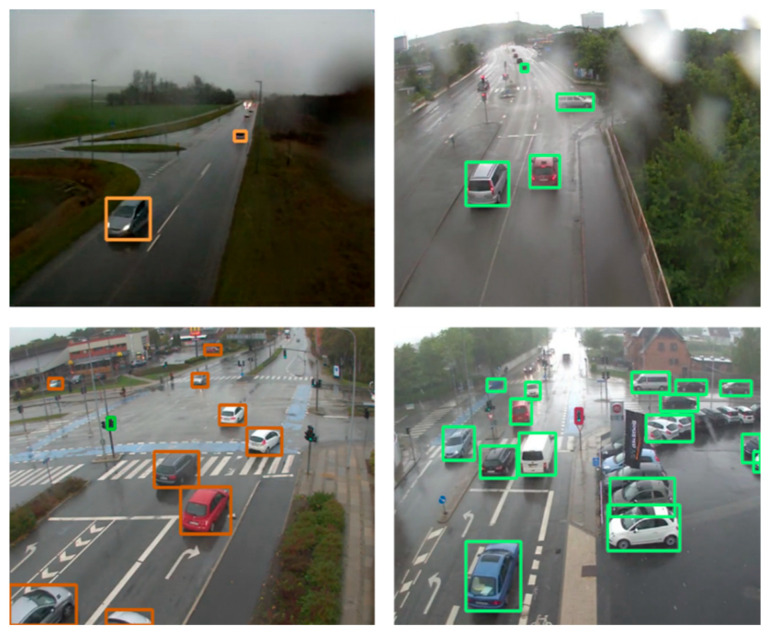
The result of detection.

In order to meet the detection speed as well as to obtain accurate detection results on large-scale datasets, yolov5 has been used as a detection tool. yolov5 uses CSPDarknet53 as the backbone and adds mosaic data enhancement and adaptive anchor frame calculation and other calculations on the input side, which greatly enriches the detection dataset. In particular, the mosaic data enhancement random scaling enhances many small targets, which improves the robustness of the network. Meanwhile, the FPN + PAN [43,44] structure is applied in the network structure, and a bottom-up feature pyramid is added after the traditional FPN layer. The FPN layer conveys strong semantic features from the top down, while the feature pyramid conveys strong localization features from the bottom up to achieve the purpose of parameter aggregation from different backbone layers to different detection layers, develops a feature map with less information loss, and enhances the detection capability of the network. Figure 8 shows video images from the AAU dataset are used to test the vehicle detection efficiency, which contains a total of 25 min of video information and 30,000 images of traffic flow with 640 × 480 resolution that are input into yolov5.

**F.**  **Stage 4: Traffic flow parameter estimation**

Although the virtual coil method has faster detection speed and less memory loss, it is rarely used for estimating traffic flow parameters in snowy weather because of the errors it generates by mistaking snow particles passing through the area for vehicles. There are two advantages of using the virtual coil method for snowfall traffic flow video images after snow removal: (1) the snow-removal model will occupy most of the memory resources, so using the virtual coil method can save memory and improve the model’s running speed; (2) the accuracy of parameter estimation by the virtual coil method can reflect the performance of the snow-removal model from the side.

The virtual coil method uses virtual induction coils instead of real induction coils and works similarly to an in-ground coil detector. The positions of 2 detection coils are defined on the image perpendicular to the road direction, and the system determines the vehicle passing by the grayscale change in the detection coils. From the number of interval frames P between the two coils before and after the vehicle passing, the distance L between the two coils in reality, and the distance S between the second coil and the stop line, the current vehicle speed and the time to reach the line can be estimated. The advantages of this method are simple operation, short time consumption, and the ability to complete speed and volume estimation in real time. The specific steps of the algorithm are as follows:

- Determine the position, size, and tilt angle of the two virtual coils, ensure that there are no vehicles in the virtual coils of the first frame sequence, and label the virtual coils in the video sequence.
- Calculate the average grayscale value of the first frame sequence in the 2 virtual coils, noted as a, b.
- Calculate the average grayscale values of the video frames in the 2 virtual coils one by one, noted as m, n, and compare them with the calculated results of the first frame a, b. When |m-a| is greater than a certain threshold, the first coil is judged to have a vehicle passing and the serial number i of the current frame is recorded, and when |n-b| is greater than a certain threshold, the second coil is judged to have a vehicle passing and the serial number j of the current frame is recorded.
- When the vehicle passes the second virtual coil, the speed of the vehicle and the time to reach the line can be estimated by i, j, the spacing of the two virtual coils in the actual reality, the spacing between the second virtual coil and the stop line, and the frame rate of the video. At the same time, each time a vehicle passes the second virtual coil, the value of the counter is increased by 1, which is used to count the traffic volume.

When the background contains more snow particles and streaks, the change in grayscale value near the coil caused by snowfall can lead to misjudgment of vehicle speed. After applying the snow-removal model proposed in this paper, a clean background image can then be successfully used for vehicle speed detection using the virtual coil method.

## 4. Experimental Results

**A.**  **Vehicle detection and snow-removal model evaluation results**

To analyze and test the performance of the proposed framework in this paper, the final model is tested on the AAU dataset. Also, the model is compared with the original image, DeSnowNet, the de-snow particle-only model, and the de-snow streak-only model on the same dataset.

The de-snow particle-only model and the de-snow streak-only model were built in python 3.8 with tensorflow 2.0, and the models were trained using the CSD dataset and the Snow100K dataset. The size of the input image is 640 × 480, the batch size is set to 32, and the number of iterations is 1500. Meanwhile, an independent program is used to test and evaluate the model in this paper. The program first converts the video files into image files frame by frame, reads the video frames one by one as the input, then runs the corresponding snow-removal models, and finally converts the output images into video files again, using yolov5 and the virtual coil method to identify and detect the speed of traffic flow. All the snow-removal models were tested using the same AAU dataset with an image resolution of 640 × 480.

Out of 2300 test samples, a total of 2100 are vehicle samples and 200 are traffic signals. The detailed accuracy rates of each method are shown in Table 1. Since each different snow-removal model uses yolov5 for target detection after snow removal, which means that there is no significant difference in the recall of the detection results of different snow-removal models (yolov5 itself has better vehicle recognition capability), this paper mainly uses the accuracy rate as an index to analyze the snow-removal capability of different snow-removal methods. In clear weather, yolov5 detects vehicles with an accuracy of 90%. This means that the upper limit of vehicle detection accuracy after snow-containing image de-snowing is about 90%.

In this paper, the study examined the effect of snow modeling in light snow (snowfall of 0–1 inch) and moderate snow (snowfall of 1–2 inches) weather conditions, respectively. The results, presented in Table 1, provide insights into the outcomes of the investigation.

From Table 1, it can be seen that the performance of different de-snowing models under light snow is not much different and all of them have high performance, which is due to the low snowfall in light snow, which causes less interference in vehicle detection and enables high vehicle detection accuracy (87.1%), even without using the de-snowing model. At the same time, it can be seen that the accuracy of vehicle detection using the original image is only 71.6% under moderate snowfall, which is 18.4% different from the accuracy of detection by yolov5 under a clear sky, indicating that the impact of snow on the accuracy of traffic flow detection is about 18.4%. In all the detection results, the detection accuracy obtained by using only the snow streak removal model is instead lower than that obtained by using the original image. This is because snow streaks tend to occupy a larger number of pixels in the image, and using only the snow streak removal model may cause some vehicles or traffic signs to be recognized as snow streaks, leading to a decrease in the detection accuracy. Of course, this is also related to the environment used for detection; in snowfall environments, snow particles tend to appear more frequently than snow streaks, so using only the snow particle removal model can effectively improve the accuracy of vehicle detection. The framework used in this paper has the highest detection accuracy, and compared to the original image, the framework used in this paper results in an 8.6% improvement under moderate snowfall in traffic flow detection accuracy. Compared to the Desnownet snow-removal model, the traffic flow detection accuracy is improved by 5.8%.

A comparison of the different model run times is shown in Table 2. A free GPU provided by Google Colab was utilized to compare the execution time of the snow-removal model employed in this research paper with other models constructed using deep learning algorithms. The same 500 snow-containing images were used as inputs to the different models to obtain the average processing time per image for each model. The results show that the snow-removal model proposed in this paper can effectively improve snow-removal efficiency compared with other current deep learning models.

**B.**  **Traffic flow parameter evaluation results**

**Figure 9:**
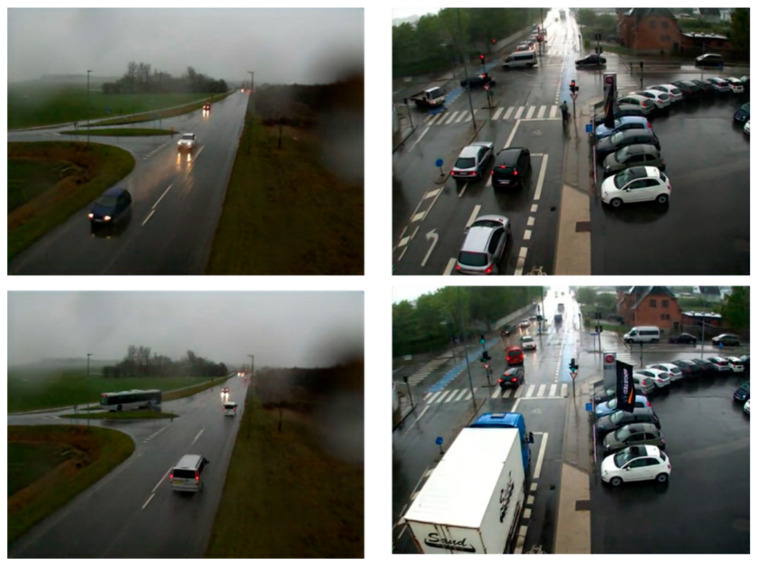
The video frame images (video 1 on the left shows traffic flow under moderate snow, and video 2 on the right shows traffic flow under light snow).

In the test, a total of two five-minute video data samples were taken for a total of ten minutes. Then, 40 consecutive vehicles were chosen from each of the two videos for the purpose of speed estimation. It should be noted that the video dataset used for testing was not used in the training of any model. Video 1 was shot on a city road in moderate snow, monitoring a smoothly flowing intersection. Video 2 was taken on a city road in light snow, monitoring a smoothly congested intersection. The test videos are all surveillance videos without background rotation and vibration. Figure 9 shows some random video windows in both videos (all have been processed by the snow-removal model). The speed of forty vehicles was determined for each video, and the values are listed in Table 3. The average speed of the traffic flow in video 1 is 26.03 mph, which is a reasonable speed for traffic flow under moderate snow. The average speed of traffic flow in video 2 is 29.35 mph, which shows that the speed of traffic flow under light snow is higher than the speed of traffic flow under moderate snow, which is also consistent with the conclusion of the study on snowfall and traffic flow speed.

**C.**  **System performance evaluation**

To verify the validity of the traffic flow parameter estimation, speed was chosen as the evaluation criterion in this paper. The ground truth speed is measured by an on-screen pixel measurement tool, and the speed of individual vehicles is measured by five consecutive vehicle frames. In general, manual measurement errors can be significant if the frame interval is too small, and the resolution of the data can be significantly degraded if the frame interval is too large. In the video used in this paper, the five frames last for approximately 0.2 s, during which the traffic speed values can be recognized as constant. Therefore, the detected speed is averaged over every five consecutive frames and is used in the accuracy analysis of the speed. Ground truth vehicle data are then collected by manual frame-by-frame counting.

**Figure 10:**
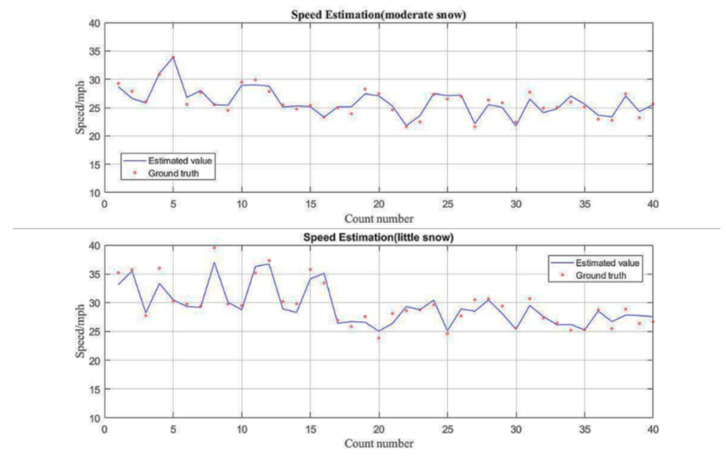
The speed estimation result.

Figure 10 shows the comparison of estimated speed and ground truth speed for the two cases. The accuracy and other detailed information are shown in Table 3. Video 2 has a high accuracy of 97.2% for the speed estimation; it can be seen that the accuracy of video 2 is higher than that of video 1. The videos were examined individually, revealing the inclusion of a greater number of trucks and sparser snow particles in video 1. Consequently, the accuracy of speed estimation in video 1 was diminished. However, in general, the proposed framework in this paper achieved good estimation performance in both light and moderate snow conditions.

The speed estimation strongly depends on the performance of the snow-removal model. This is because when the snow particles and snow streaks are not completely removed, the changes in image grayscale values from the remaining snow in the image can cause the detector to mistake vehicles, which can lead to anomalies in the traffic flow parameters. In the framework used in this paper, the snow-removal model can effectively remove the residual snow in the image, so that good parameter detection accuracy can be obtained.

Although the framework proposed in this paper achieves good traffic flow parameter estimation under both light and moderate snowfall, it should be noted that the accuracy of the model decreases as the snowfall rises, which is because the density of snow particles under heavy snowfall is much higher than that of light-to-moderate snowfall, and it is difficult for the existing snowfall model to remove them.

## 5. Conclusions

In this paper, a framework for identifying and extracting traffic flow parameters from snow-containing images has been proposed, which consists of four steps. In the first two stages, a DTCWT deep learning network and a negative mapping network are trained to remove snow particles and snow streaks from snow-containing images, respectively. DTCWT can extract snow particles with different angles and sizes from the images, which improves the snow particle removal performance of the model, and the negative mapping network with image sparsity can effectively remove snow streaks from the images. In the third stage, the yolov5 network is used to identify the vehicles in the images. In the fourth stage, the virtual coil method is used to extract the speed and traffic volume of the traffic in the video. The experimental results show that the proposed framework in this paper has excellent performance in light and moderate snowy weather and can effectively remove snow particles and streaks from images and extract traffic flow parameters.

Future work will focus on the following areas. Firstly, more video data of traffic flow under heavy snow will be collected and used in the training process of the model to improve the performance of the model under heavy snow. Secondly, the performance of the proposed framework will be further evaluated over longer monitoring times and in more complex traffic scenarios, such as low video quality, different photo conditions, etc. Thirdly, the study endeavors to tackle the identification of traffic flow and the extraction of its parameters under snowy weather conditions. Subsequently, future investigations are intended to delve deeper into examining the operational attributes of traffic flow during snowy weather.

## Figures and Tables

**Table 1:** Detection performance evaluation (a comparison of the results under light and moderate snowfall).

		Right	False	Accuracy
Little snow	Snow image	2003	297	0.871
	Desnownet	2012	288	0.875
	Particle only	2019	281	0.878
	Streak only	2005	295	0.872
	Framework	2029	271	0.882
Moderate snow	Snow image	1647	653	0.716
	Desnownet	1712	588	0.744
	Particle only	1691	609	0.735
	Streak only	1521	779	0.661
	Framework	1852	448	0.802

**Table 2:** Comparison of run time.

	DesnowNet	JSTASR [31]	Our
Run Time (s)	2.12	1.78	0.45

**Table 3:** Estimated traffic flow speed.

	Video 1	Video 2
Number of vehicles	40	40
Image resolution	640 × 480	640 × 480
Weather condition	Moderate snow	Little snow
Average speed estimation (mph)	26.03	29.35
Accuracy (%)	95.1	97.2
Video frame rate (fps)	20	20

## Data Availability

The data presented in this study are available from the corresponding author upon request due to privacy concerns.
